# Development and spontaneous closure of a secondary macular hole associated with submacular hemorrhage due to polypoidal choroidal vasculopathy: a case report

**DOI:** 10.1186/s12886-020-01370-8

**Published:** 2020-03-17

**Authors:** Minami Chino, Yuji Yoshikawa, Junji Kanno, Takamitsu Nagashima, Yu Sakaki, Takeshi Katsumoto, Masayuki Shibuya, Takuhei Shoji, Jun Makita, Kei Shinoda

**Affiliations:** 1 Department of Ophthalmology, Faculty of Medicine, Saitama Medical University, 38 Moro-Hongo Moroyama-machi, Iruma-gun, Saitama, 350-0495 Japan; 2 Kamishirane Hospital, 2-65-1, Kamishirane, Yokohama, Kanagawa 241-0002 Japan

**Keywords:** Macular hole, Retinal displacement, Spontaneous closure, Submacular hemorrhage

## Abstract

**Background:**

Macular hole (MH) is a retinal break in the fovea involving partial or complete dehiscence of the neural retinal layers affecting the visual quality by decreasing visual acuity (VA) and visual deformation. We describe a case of secondary MH associated with submacular hemorrhage (SMH) due to polypoidal choroidal vasculopathy (PCV), which showed spontaneous closure.

**Case presentation:**

A 67-year-old man developed decreased VA in his right eye due to an SMH. The VA was 20/50, and monthly intravitreal injection of aflibercept was administered three times. The SMH gradually decreased, and 10 months later the external limiting membrane was found to be perforated, resulting in MH. The old clot disappeared, and the MH remained for 10 months. Twenty-three months later, serous retinal detachment (SRD) involving the macula appeared and the MH had disappeared. SRD gradually disappeared, and macular configuration recovered. VA gradually improved and became 20/20 38 months later.

**Conclusion:**

Dynamic change of the ultrastructure in an unusual case of secondary-developed and spontaneously closed MH was clearly observed. Although the mechanism was unknown, the small diameter size and exudative PCV are thought to have contributed to the closure.

## Background

Macular hole (MH) is a retinal break in the fovea involving partial or complete dehiscence of the neural retinal layers affecting the visual quality by decreasing visual acuity (VA) and visual deformation [1]. Formation of an idiopathic MH has been attributed to mechanical forces exerting tangential traction at the vitreomacular interface. Secondary MH may develop in association with trauma [2], macular edema [3], retinal detachment [4], choroidal neovascularization [5–7], or submacular hemorrhage (SMH) [8–10]. Spontaneous closure is a rare event [7, 10–16]. We present a rare case of the development of secondary MH and spontaneous closure of MH associated with SMH due to polypoidal choroidal vasculopathy (PCV).

## Case presentation

**Fig. 1:**
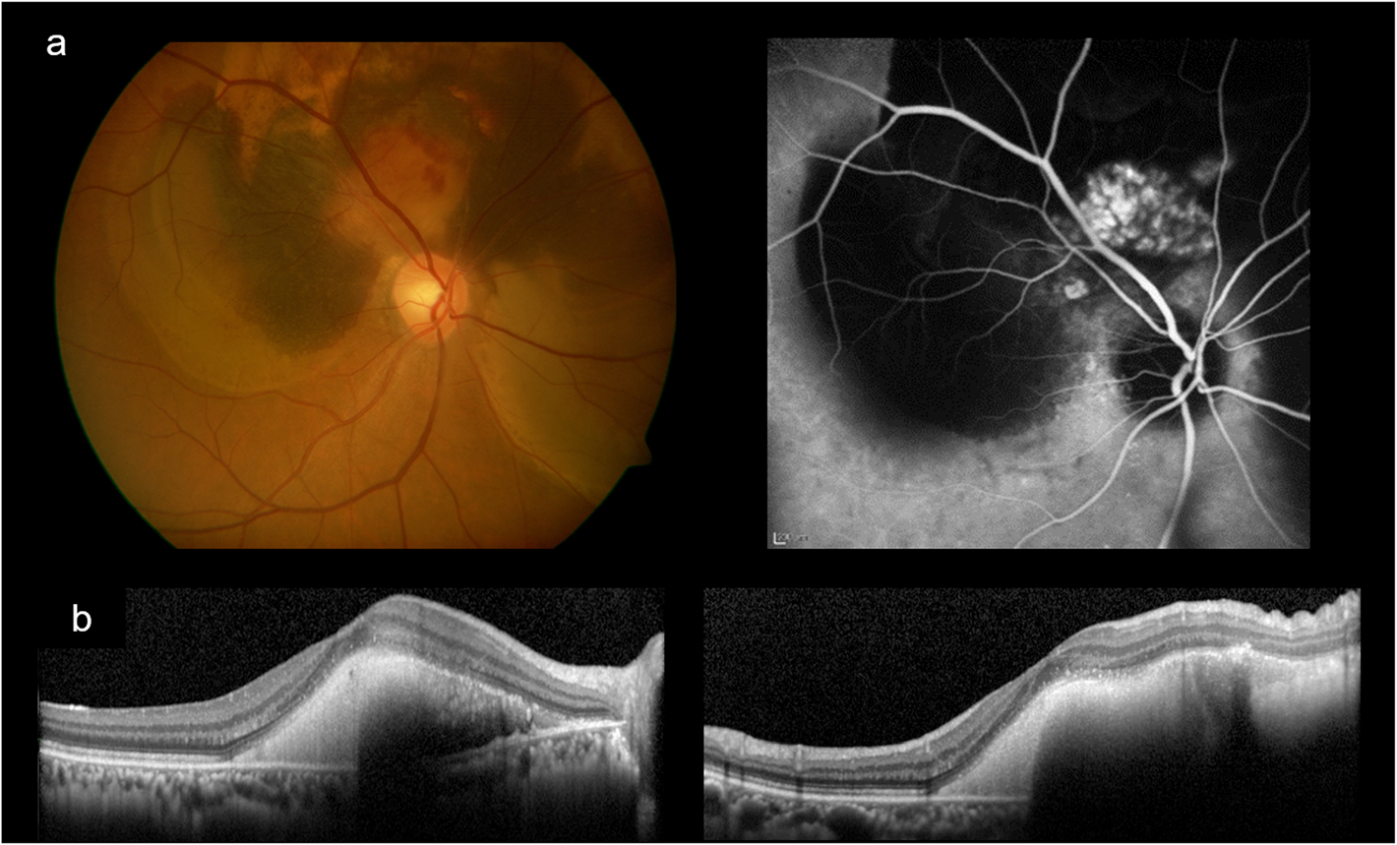
Fundus appearance and angiographic findings at onset. **a** Fundus photograph showing subretinal hemorrhage that was centered superior nasal to the optic disc and overhung the macula. There seemed to be different regions of hemorrhage, colored yellow, bright red, and dark red. There was also serous retinal detachment surrounding the optic disc superiorly at 270 degrees. Indocyanine green fundus angiography showing leakage from multiple tufted vessels superior and superior nasal to the optic disc creating the hyperfluorescent area in the image. **b** Optical coherence tomography showed submacular hemorrhage without macular hole. (right; horizontal scan, left; vertical scan)

**Fig. 2:**
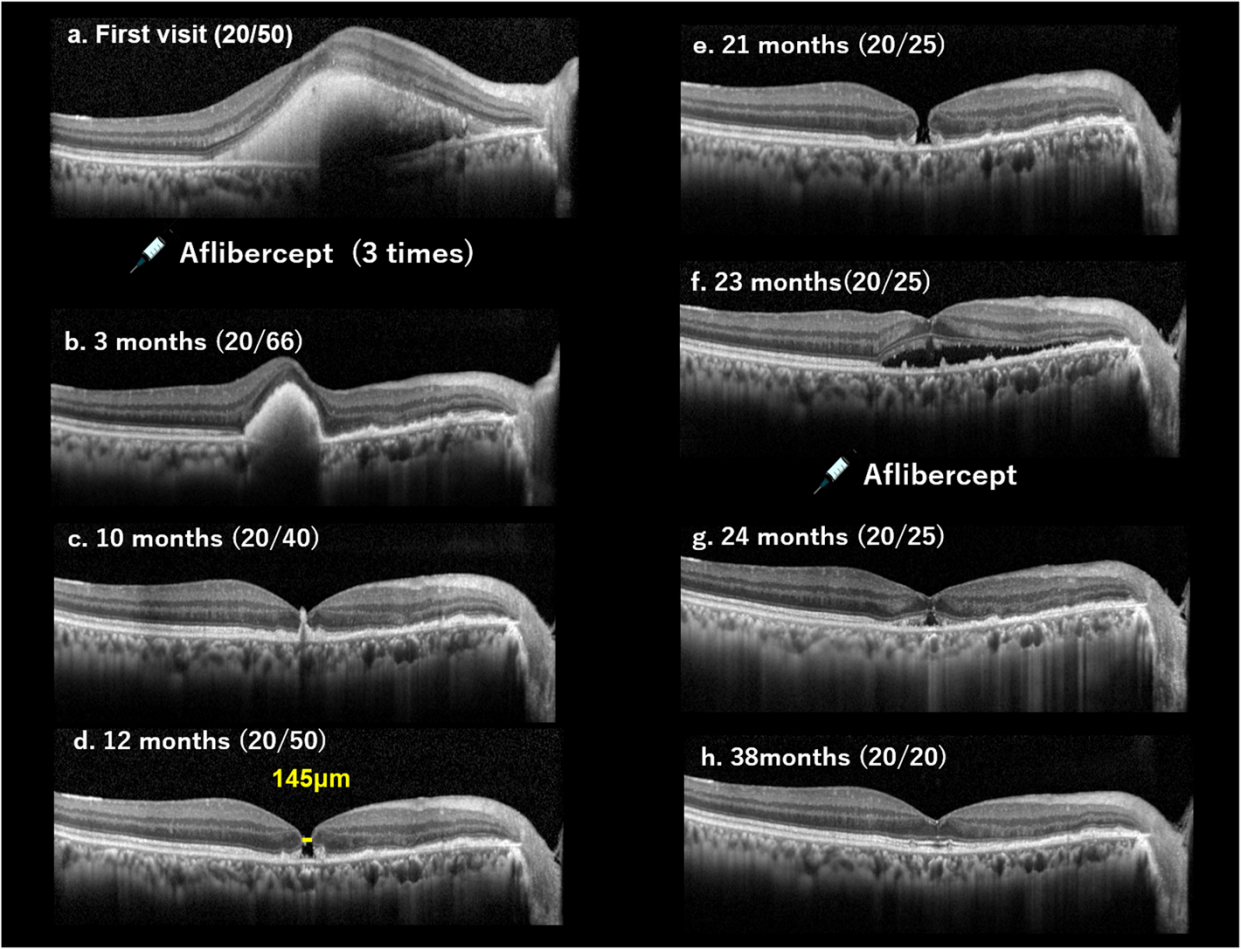
Macular structure monitoring by optical coherence tomography. Optical coherence tomography (OCT) images showing the progression of macular hole formation and closure. **a** At first visit, OCT shows dense submacular hemorrhage (SMH). The visual acuity (VA) was 20/50. **b** Three months later, when monthly intravitreal injection of aflibercept (IVA) was administered three times, the SMH decreased. The VA was 20/66. **c** Ten months later, SMH further decreased and the external limiting membrane (ELM) was found to be perforated. The VA was 20/40. **d** Twelve months later, MH was observed. The minimum diameter of MH was 145 μm. The VA was 20/50. **e** Twenty-one months later, the MH had remained for 10 months. The VA increased to 20/25. **f** Twenty-three months later, serous retinal detachment (SRD) involving the macula appeared, and the MH disappeared. The VA was 20/25 and IVA was performed again. **g** Twenty-four months later, the SRD was seen to be gradually disappearing, and macular configuration had recovered. The VA was 20/25. **h** Thirty-eight months later, the SRD had disappeared, and the macular ultrastructure had approached to sound. The VA was 20/20

**Fig. 3:**
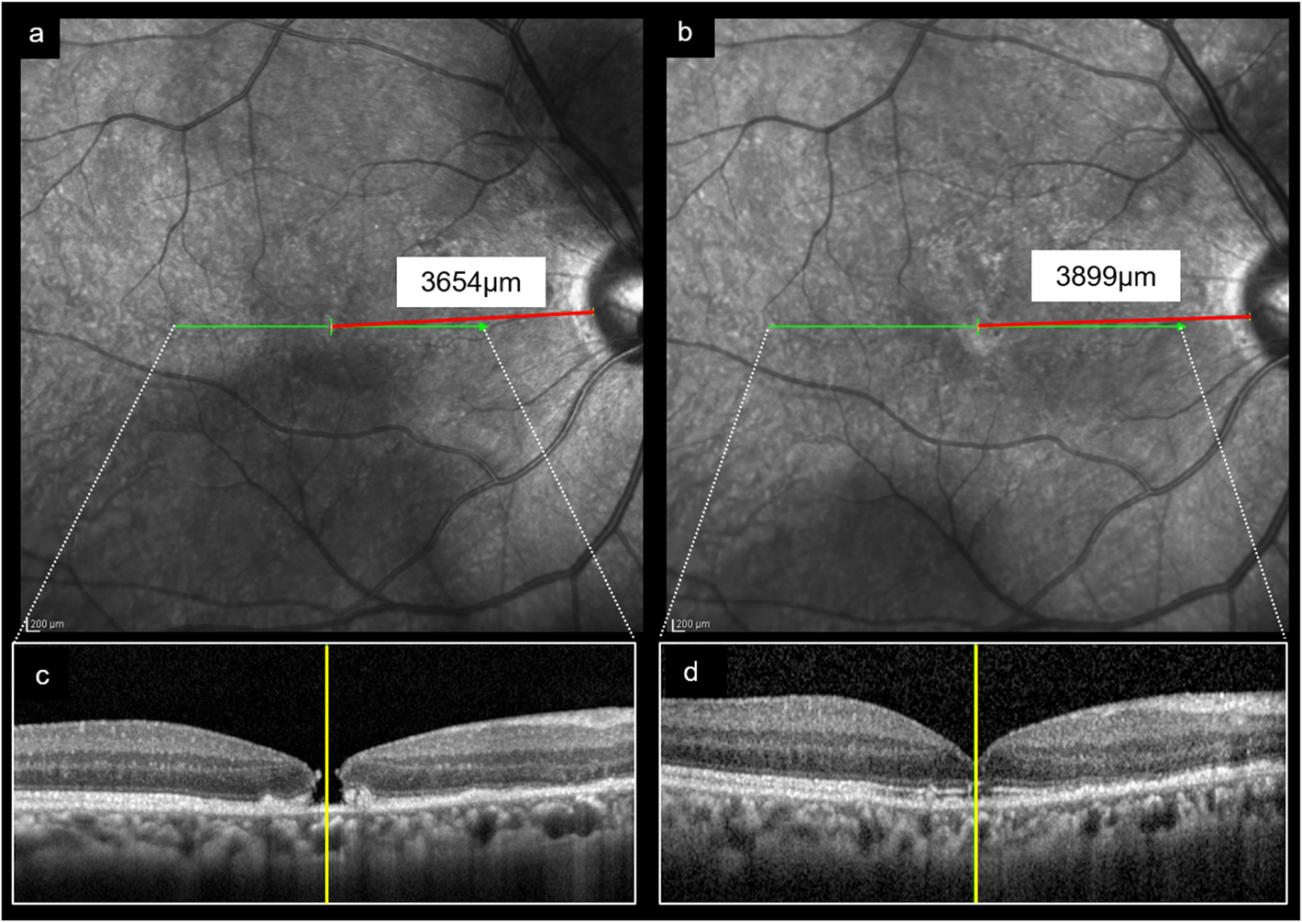
Distance between the fovea and optic disc margin before and after macular hole closure. a and b; Fundus image showing the scanning line (indicated by green arrow) and the distance between the temporal disc margin and fovea (indicated by red line). The location of the fovea was determined in the B scan image as shown below . c and d; B scan image of the macula showing the location of the fovea (indicated by the yellow line). The distance between the fovea, which was determined by the B scan image, and the temporal optic disc margin, which was determined by large vessels, was measured on optical coherence tomography images. The distance before macular hole (MH) closure was 3654 μm (**a**). After MH closure, the distance was 3899 μm (**b**). There was no appearance of foveal displacement toward the optic disc after MH closure

A 67-year-old man developed decreased VA in his right eye due to SMH. Fundus examination showed vitreoretinal separation and subretinal hemorrhage centering superior nasal to the optic disc and hanging over the macula. Fundus angiography revealed PCV (Fig. 1). The VA was 20/50 and monthly intravitreal injection of aflibercept (IVA) was administered three times. The SMH gradually decreased, and 10 months later the external limiting membrane (ELM) was found to be perforated, resulting in MH at 12 months after the initial visit. The minimum diameter of MH was 145 μm. Because the patient refused to undergo surgery, the MH remained for 10 months after MH developed. Twenty-three months after the initial visit, serous retinal detachment (SRD) involving the macula appeared and the MH disappeared. Fundus angiography showed moderate leakage from PCV, and IVA was performed again. Twenty-four months after the initial visit, the SRD gradually disappeared, and macular configuration recovered. During the course of the treatment, VA gradually improved and became 20/20 38 months after the initial visit (Fig. 2). The distance between the fovea and the optic disc margin before MH closure was 3654 μm. After MH closure, the distance was 3899 μm. There was no appearance of foveal displacement toward the optic disc after MH closure (Fig. 3).

## Discussion and conclusions

We presented a rare case of development of secondary MH and spontaneous closure associated with SMH due to PCV. Two unusual findings were observed: late onset of secondary MH associated with SMH due to PCV and spontaneous closure and return of excellent VA.

Several macular pathologies have been reported that may develop secondary MH such as cystoid macular edema, subretinal fluid due to rhegmatogenous retinal detachment, and SMH [2–10]. In most of the secondary MH associated with SMH, the SMH was caused by rupture of a retinal arterial macroaneurysm (RAM) [17–19]. There are several reports of secondary MH associated with choroidal neovascularization, but they developed after absorption of submacular fluid, not SMH [5–7]. To the best of our knowledge, this is the first report of a case of secondary MH associated with SMH due to PCV.

In a case of SMH due to RAM, it was considered that secondary MH was caused by rapid increase in intraretinal pressure (early onset) or by retinal degeneration (late onset) [18]. Sagara et al. reported that the presence of subretinal and sub-internal limiting membrane (ILM) hemorrhages after rupture of a RAM might contribute to formation of an MH [17].

Experimental studies on animals have also shown relationships between SMH and severe retinal degeneration [20, 21]. Fibrin-mediated retinal damage may be an important cause of retinal degeneration related to subretinal hemorrhage [22]. Another explanation might be that retinal inflammation was associated with the pathogenesis of MH formation as indicated by Hayashi et al. [13].

Several mechanisms for MH formation have been previously hypothesized: anteroposterior and tangential traction caused by vitreous incarceration in a needle entry site [5, 6], exacerbated tangential traction from contraction of the vascular membrane [5, 6], and critical reduction in the cell–cell attachments of the retinal pigment epithelium following anti-vascular endothelial growth factor treatment [23].

In the current case, perifoveal vitreous detachment was seen on the first visit, and there was no change in the vitreoretinal interface in the course of MH formation. Thereafter, the MH appeared 12 months after the patient’s first visit. Thus, we hypothesized that the process that created this MH (late onset) was retinal degeneration or retinal injury by SMH.

An outstanding feature is that the patient in our case showed spontaneous MH closure without surgery and achieved excellent VA recovery. A previous report showed that a secondary MH, which was associated with intravitreal injection for choroidal neovascularization (CNV), achieved closure after surgery [5]. Another study reported closure of MH that developed in association with SMH due to CNV or RAM achieved by vitrectomy with ILM peeling and tamponade [8, 9].

Several case studies have also reported spontaneous closure of MH and have attributed the cause to vitreomacular traction relief over time (vitreoretinal separation), formation of bridging intraretinal tissue (due to epiretinal membrane, extension and proliferation of glial cells, or collagen secreted by glial cells), absorption of cystic space, sharp edge, inflammation, and small size of MH [1, 12, 16, 24]. Su et al. reported spontaneous closure of full thickness MH with age-related macular degeneration and suggested that the exudative process or secondary healing that stimulated adjacent glial cells contributed to MH closure [7]. Oliver et al. and Wang et al. reported that macular detachment, which released the retina from the retinal pigment epithelium (RPE), might have prompted approximation of the MH edge, thus facilitated MH closure [25, 26]. In the current case, SRD, which appeared in the macular area, probably facilitated approximation of the MH edges by releasing the MH edge from the RPE. Therefore, the exudative change and accumulation of submacular fluid might have contributed to the spontaneous MH closure. Moreover, in the case of traumatic MH, small size and absence of intraretinal cysts were predictive factors of spontaneous closure of MH [24]. The authors described that the presence of intraretinal cysts might be an indicator of ILM traction. In the current case, the MH diameter was small and there was no appearance of intraretinal cysts, both of which might be associated with spontaneous closure.

Kawano et al. reported that successful closure of MH by vitrectomy with ILM peeling and gas tamponade led to a displacement of the center of the macula and that eyes with spontaneous MH closure without surgery had no significant foveal displacement [14]. In the current case, there was no appearance of foveal displacement toward the optic disc after MH closure. Thus, we hypothesized that several factors, such as the small diameter of the MH, absence of intraretinal cysts, and associated presence of exudation and macular detachment with PCV, contributed to the spontaneous MH closure but that closure was not related to the release of macular traction.

In conclusion, we presented a rare case of secondary MH and spontaneous closure, which was associated with SMH and PCV. Imaging analysis clearly illustrated the dynamic course of the formation and closing phase of the MH. Although the precise mechanism of closure was unknown, the small size of the MH and exudation due to PCV are thought to have contributed to it.

## Data Availability

Not applicable.

## Abbreviations

CNV: Choroidal neovascularization
ELM: External limiting membrane
ILM: Internal limiting membrane
IVA: Intravitreal aflibercept injection
MH: Macular hole
PCV: Polypoidal choroidal vasculopathy
RAM: Retinal arterial macroaneurysm
SMH: Submacular hemorrhage
SRD: Serous retinal detachment
VA: Visual acuity
